# Inactivation of the Rgg2 Transcriptional Regulator Ablates the Virulence of *Streptococcus pyogenes*

**DOI:** 10.1371/journal.pone.0114784

**Published:** 2014-12-08

**Authors:** Anastasia A. Zutkis, Srivishnupriya Anbalagan, Michael S. Chaussee, Alexander V. Dmitriev

**Affiliations:** 1 Department of Molecular Microbiology, Institute of Experimental Medicine, Saint-Petersburg, Russia; 2 Division of Basic Biomedical Sciences, Sanford School of Medicine of the University of South Dakota, Vermillion, South Dakota, United States of America; 3 Saint-Petersburg State University, Saint-Petersburg, Russia; University of Kansas Medical Center, United States of America

## Abstract

*Streptococcus pyogenes* adapts to different niches encountered in the human host via the activity of numerous regulatory proteins including the Rgg family of transcriptional regulators. The *S. pyogenes* chromosome encodes four Rgg paralogues designated Rgg1 (RopB), Rgg2 (MutR), Rgg3, and Rgg4 (ComR). In order to understand the role of the Rgg2 protein in the regulation of metabolic and virulence-associated properties of *S. pyogenes*, the *rgg2* gene was inactivated in the M1 serotype strain SF370. Inactivation of *rgg2* increased the growth yield of *S. pyogenes* in THY broth, increased biofilm formation, and increased production of SIC, which is an important virulence factor that inhibits complement mediated lysis. To identify Rgg2-regulated genes, the transcriptomes of SF370 and the *rgg2* mutant strains were compared in the middle-exponential and post-exponential phases of growth. Rgg2 was found to control the expression of dozens of genes primarily in the exponential phase of growth, including genes associated with virulence (*sse*, *scpA*, *slo*, *nga*, *mf*-3), DNA transformation, and nucleotide metabolism. Inactivation of *rgg2* decreased the ability of *S. pyogenes* to adhere to epithelial cells. In addition, the mutant strain was more sensitive to killing when incubated with human blood and avirulent in a murine bacteremia model. Finally, inoculation of mice with the avirulent *rgg2* mutant of *S. pyogenes* SF370 conferred complete protection to mice subsequently challenged with the wild-type strain. Restoration of an intact *rgg2* gene in mutant strain restored the wild-type phenotypes. Overall, the results demonstrate that Rgg2 is an important regulatory protein in *S. pyogenes* involved in controlling genes associated with both metabolism and virulence.

## Introduction

*Streptococcus pyogenes* adapts to different niches encountered in the human host such as the pharynx, skin, and blood. To do so, it must sense various environmental cues and respond with appropriate changes in gene expression [1]. Transcriptional regulatory proteins of the Rgg family contribute to this ability.

The *S. pyogenes* chromosome encodes four Rgg paralogues designated Rgg1, also known as RopB (Spy_2042), Rgg2, also known as MutR (SPy_0496), Rgg3 (Spy_0533), and Rgg4, also known as ComR (Spy_0037) [2]–[4]. Typically, one or more genes encoding small hydrophobic peptides are adjacent to the *rgg* genes in the chromosome [3], [5], [6]. The peptides are secreted from the bacterial cell and subsequently imported where they are thought to bind to Rgg regulators thereby altering the specificity of DNA binding and gene expression.

One theme arising from the study of Rgg regulators of various species (*Streptococcus pneumoniae, Streptococcus mutans, Streptococcus thermophilus, Streptococcus salivarius, Streptococcus infantarius* and *Streptococcus macedonicus*) is that they are important in regulating the expression of genes involved in the functionally related processes of biofilm formation and dispersal and competence for DNA transformation [7]–[10]. Moreover, the various paralogues in *S. pyogenes* appear to interact, sometimes in an opposing manner, to control these processes. For example, inactivation of *rgg1* or *rgg3* in strain NZ131 increases biofilm formation while inactivation of *rgg2* decreases biofilm formation in an *rgg3* mutant background [3].

The purpose of this study was to identify changes in gene expression associated with *rgg2* inactivation and determine if Rgg2-dependent regulation of gene expression contributes to the virulence of *S. pyogenes*. We found that *rgg2* inactivation had the most pronounced effect on gene expression in the exponential phase of growth compared to the post-exponential phase. We also found that Rgg2 repressed virulence-associated genes encoding secreted proteins and expression of the enzymes that synthesize the hyaluronic acid capsule; however, inactivation of *rgg2* abolished virulence in a murine model of bacteremia and the ability of *S. pyogenes* to grow in human blood.

## Materials and Methods

### Bacterial strains, plasmids, and growth conditions

The wild-type *S. pyogenes* strain SF370 (serotype M1) and 29 additional clinical isolates of different serotypes used in this study were previously described [4], [11], [12]. *S. pyogenes* was grown at 37°C without agitation with Todd-Hewitt broth (Becton Dickinson, USA) containing 0.2% (wt/vol) yeast extract (THY) or chemically defined media (CDM) [13] with or without the addition of 1%_f.c_. neopeptone. *Escherichia coli* was grown with LB medium at 37°C with agitation or on LB agar plates. When appropriate, erythromycin (Em; 2.5 µg/ml for *S. pyogenes* and 200 µg/ml for *E. coli*) or kanamycin (Kn; 50 µg/ml for *E. coli* and 500 µg/ml for *S. pyogenes*) was added to the media.

### Routine genetic techniques

Chromosomal DNA was isolated by phenol-chloroform extraction. Plasmid DNA was isolated and purified using AxyPrep Plasmid Miniprep Kit or AxyPrep Plasmid Midiprep Kit (Axygen Biosciences, USA) according to the manufacturer's instructions. PCR was carried out with *Taq* polymerase with initial denaturation of 2 min at 94°C followed by 30 cycles of amplification steps of 30 sec at 94°C, 1 min at 52°C, and 1 min at 72°C. PCR products were purified with AxyPrep DNA Gel Extraction Kit (Axygen Biosciences). DNA sequencing was performed employing ABI 3100 automated DNA sequencer using the Big-Dye Terminator Kit (Applied Biosystems, USA).

### Insertional inactivation of *rgg2*

The *rgg2* gene was amplified using the primers Rgg2-1 (5′ - CAT GAC TGT CTC CTT TCT GAT TTT C - 3′) and Rgg2+1 (5′ - CCG TTA TTT AAA GGA CAG CTA GAC C - 3′). The PCR product was digested with *Sac*I and *Pst*I resulting in a 520 bp internal fragment that was gel purified and cloned into the vector pVA891-2 [14]. Following transformation of *E. coli* strain DH5α (Gibco-BRL, USA) the recombinant plasmid, designated pVA891-2[rgg2], was isolated and used to transform *S. pyogenes* strain SF370. Transformants were selected with agar plates containing Em and insertional inactivation of *rgg2* was verified by PCR and sequencing with the primers Rgg2-1, Rgg2+1, 40/1 (5′ - AGG AGG GAC AGC TGG ATA TTA CG – 3′), and 40/2 (5′ - TCC CAT TTA GCC GTC ATT TCA G - 3′).

### Restoration of the *rgg2* gene in mutant strain

The chromosomal restoration of the *rgg2* gene in mutant strain was done using the protocol recently described [15]. To do so, the *E. coli* – streptococcus shuttle vector pMSP3535Va (Kn^r^) was propagated in *E. coli*. As expected, in some of the transformants the pMSP3535Va had a reduced size due to the loss of the Gram-positive origin of replication, as previously reported [16]. This derivative plasmid was isolated, purified, and designated pMSP3535Va-der. Subsequently, the entire *rgg2* of strain SF370 was cloned into pMSP3535Va-der. The primers Rgg2full-F (5′ – CGG GAT CC C GAT GGA AAA AGA ACT C - 3′) and Rgg2full-R (5′ – CCG AAT TC C GAA CAC ATC TGA TAG AAA G - 3′) containing *Bam*HI and *Eco*RI restriction sites, which are underlined, were used to amplify the *rgg2* open reading frame. The PCR product was digested with *Bam*HI and *Eco*RI, and ligated with *Bam*HI-*Eco*RI digested pMSP3535Va-der, and the ligation mixture used to transform *E. coli* strain DH5α. Kn^R^ clones were selected and a 6.7 kb recombinant plasmid designated prgg2, was isolated. prgg2 was used to transform the *S. pyogenes rgg2* mutant strain using Gene Pulser Xcell Electroporation System (Bio-Rad Laboratories, USA), as recommended by the manufacturer. Following homologous recombination, two different recombinant derivatives were identified. In one case, the prgg2 vector was integrated into the disrupted 5′ *rgg2* gene fragment, which resulted in restoration of an intact *rgg2* adjacent to its native promoter (data not shown). This complemented strain was designated SF370 *rgg2*/prgg2 and selected for further study. PCR and nucleotide sequencing with the primers Rgg2-1, Rgg2+1, 40/1, 40/2, VaFor (5′ - CCC CTG ATT CTG TGG ATA ACC GT – 3′), and VaRev (5′ - TTT CGC TAT GTA CAC CCG GTT G - 3′) were used to confirm the construction of complemented strain.

### Determination of SpeB proteolytic and DNase activities

Extracellular proteolytic activity was assessed with agar plates containing casein, as previously described [2]. DNase activity was determined using DNA-containing agar plates as described [13].

### Protein isolation and SDS-PAGE

Culture supernatant proteins from SF370 wild-type strain and *rgg2* mutant strains were isolated from 40 ml of middle-exponential phase cultures by acetone precipitation and separated by conventional SDS-PAGE.

### Biofilm assays

Biofilm formation assays were done essentially as previously described [17]. The wild-type and mutant strains were cultured with either peptide-free chemically defined media [18] or THY in 24 well plates for 24 h at 37°C in 5% CO_2_. The wells were washed three times with 200 µl of phosphate-buffered saline (PBS) and 200 µl of 0.1% (wt/vol) crystal violet was added to each well. After 30 min., the wells were washed twice with 200 µl of sterile deionized water to remove unbound crystal violet. The remaining crystal violet associated with adherent biomass was dissolved in 200 µl of 95% ethanol and the absorbance was measured at 600 nm. Four wells were used for each strain and the average value determined. The experiment was repeated four times and the mean ± standard error of the mean is reported. The Student's t-test was used to compare the mean values between the strains.

### Protein identification

Proteins of the interest were excised from SDS-PAGE gels. After trypsinization, the peptides were analyzed by mass-spectrometry in positive ion mode using maXis (Brucker Daltonics, Germany), according to protocols recommended by the manufacturer. The spectra were obtained, and the MASCOT software (www.matrixscience.com) was used to analyze peptides against the NCBI non-redundant *S. pyogenes* database.

### RNA isolation

**Figure 1:**
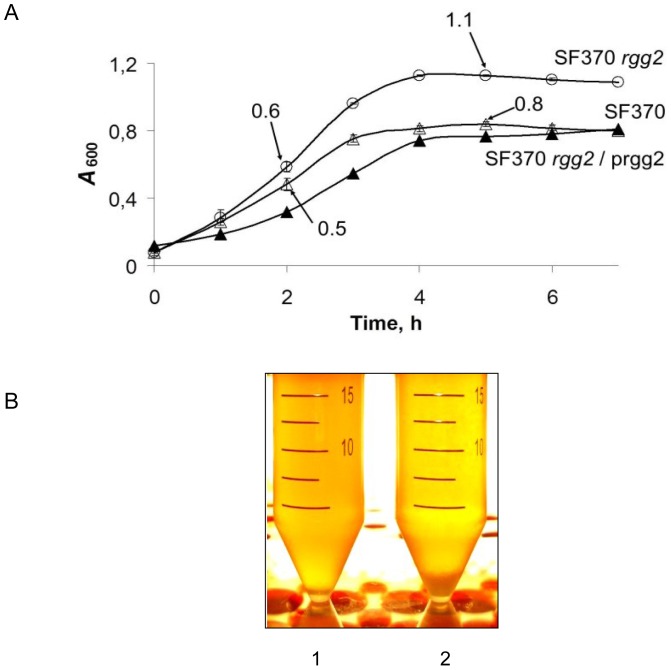
Growth of the strains under study in THY broth. Panel A. SF370 wild-type strain (Δ), *rgg2* mutant (o), and the complemented strain (▴ SF370 *rgg2*/prgg2) were cultured in THY broth. The *A*_600_ of the cultures are shown as the means and standard errors of the means (SEM) from three independent experiments. Arrows designate the time of RNA isolation. Panel B. SF370 wild-type strain and *rgg2* mutant strains were cultured in THY broth at 37°C without agitation for 3 hrs. The experiment was done in triplicate and the results reproducibly showed predominant growth of the SF370 strain (2) at the bottom of the tube compared to the *rgg2* mutant strain (1).

RNA was isolated from 40 ml cultures of *S. pyogenes* in the middle exponential and post-exponential phase corresponding to 2 hrs and 5 hrs of growth, respectively (Fig. 1) with an RNeasy Mini Kit (QIAGEN, USA), as recommended by manufacturer. The concentration and quality of the RNA was determined with an Agilent 2100 Bioanalyzer (Agilent, USA) using an RNA 6000 Nano LabChip kit (Agilent).

### Quantitative reverse transcriptase (RT)-PCR

Oligonucleotide primers used for qRT-PCR are listed in the Table 1. The primers were designed with Primer Express 2.0 software (ABI Prism, PE Biosystems, USA) and purchased from Sigma-Genosys (USA). Amplification and detection were done with the ABI Prism 7700 Sequence Detection System (PE Applied Biosystems) using Power SYBR Green RNA-to-C_T_ 1–Step Kit (PE Applied Biosystems), as recommended by the manufacturer.

**Table 1:** Oligonucleotide primers and the changes in gene transcript levels as determined with quantitative RT-PCR.

SPy no a	Gene	Description	Forward primer (5′ → 3′)	Reverse primer (5′ → 3′)	Fold change (*rgg2*/wt) b
0165	*nga*	NAD glycohydrolase	CAC CTA CAC TAA AAA ACC GCA TCA	CAA AAG TGA CCT CTG ACA AGG CTA A	7.0
0901	*pyrE*	orotate phosphoribosyltransferase	CCC TTT TAC GTG GGC ATC TG	CTT CTG GGA AAT GAG CCT TGA T	−1.5
1902	*comX*	alternate sigma factor	AGT CAA AAG CGT CAG TTC CAT AAG T	AGC CAC ATA GTC GTC TAG CCA TAA C	3.7
0037	*rgg4*	transcriptional regulator	GGA TTA GTC GCG AGG ACT TGT G	GAC CTA GTT CTA TCC GTG CTA ATT GA	2.3
1059	*ptsC*	PTS transporter	TTG GAA TGT TGG CAG CCT TT	CCA AGT GCC ATA GTG GAG CAT	−3.2
1204	*guaA*	GMP synthase	GCC ATA AAA TCA CCG CTC AAG	GCC CTC CTG ATA AAA CGA TAC CT	−400
1718	*sse*	secreted esterase	GCA GGT GGA GGT TTA GCC TTA G	CTT TGG TTG AGG GAC TGA TTC AT	194
2010	*scpA*	C5a peptidase	AAA CGA AAG AAC CTT ACC GCC	CGA CAC GCA TCA AAA GCA A	3.3

a SPy numbers are based on annotation of the SF370 *S. pyogenes* strain complete genome [4].

b Change in gene transcript level for *rgg2* mutant compared to that for the wild type.

### DNA microarray analysis

Affymetrix NimbleExpress Arrays were purchased from Affymetrix (USA) [19]. The arrays consisted of 3367 qualifiers representing 1745 predicted *S. pyogenes* ORFs, 1568 intergenic region probes, and 54 control oligos used for spike-ins. Microarray hybridization and analysis of the data was done as recently published [13]. The average signal intensity value of each gene was transformed to a log_2_ (log base 2) value. The change between two experimental conditions (*n*-fold) was calculated by taking the ratio of the signal intensity (difference of the log_2_ value) between experimental conditions. Present and absent calls were assessed and statistically significant genes (T-test; *P* value ≤0.05) were identified. All the statistical analyses were done using ArrayStar software (DNASTAR, USA). The microarray data are available through the Gene Expression Omnibus data repository via accession number GSE 57462.

### Survival assay of *S. pyogenes* in the whole human blood

Heparinized human blood (450 µl) was mixed with 450 CFUs of *S. pyogenes* in 150 µl and incubated at 37°C. The tubes were inverted every 15 min and the number of viable CFUs were determined by the plating aliquots onto solid agar media. The experimental procedures with human blood were approved by Saint-Petersburg Institute of Experimental Medicine Ethic Committee and all the individuals provided written informed consent.

### Adhesion assay of *S. pyogenes* strains to human epithelial cells

The modified protocol for analysis of adherence capacity of *S. pyogenes* to vaginal epithelial cells [20] was developed in the study. Briefly, three healthy women of 25–55 years old were donors of the epithelial cells. The cells were collected by sterile cotton swabs and washed three times with PBS and visualized with Leica DM750 microscope (Leica Microsystems, Germany). *S. pyogenes* was cultured overnight, washed with PBS, and 10^7^ CFUs were incubated with 10^5^ vaginal epithelium cells at 37°C for 30 min. The samples were visualized with Leica DM750 microscope and the number of streptococcal cells adhered to single epithelium cell was calculated. The experiments were approved by Saint-Petersburg Institute of Experimental Medicine Ethic Committee and all the donors of epithelial cells provided written informed consent.

### Ethics Statement

Outbred six-week old male mice (Rappolovo Animal Facility, Russia) were used in all the experiments. The animals were housed according to standard animal laboratory conditions. They were maintained in polycarbonate cages with stainless steel wire-bar lid, sterile bedding and free access to sterilized balanced food and water. All the experimental procedures were done according to the principles and guidelines for the care and use of laboratory animals (Russian Academy of the Medical Sciences, Russia) and were approved by Saint-Petersburg Institute of Experimental Medicine Animal Care Unit Committee, Russia (Protocol No. 3, 2011). The animals were sacrificed by CO_2_ asphyxiation and cervical dislocation, and all the efforts were done to minimize suffering.

### Murine infection model

Overnight cultures of *S. pyogenes* were harvested, suspended in PBS and plated onto agar plates to determine CFUs. The suspensions at a concentration of 1 to 5×10^8^ CFUs in 0.5 ml of PBS were prepared depending on the design of the experiment. 0.5 ml of the bacterial suspension was injected intraperitoneally into anesthetized (inhalation of isoflurane) mice. Each experimental group of animals contained between 10 and 15 mice. As control, 0.5 ml of PBS was injected into a control group of animals. Observation was done for 10 days, and the animals were monitored four times a day during the period 9:00 am–18:00 pm. The endpoints for sacrifice were defined as follows: extreme presentation of the clinical signs of infection (huddling, hunched posture, ruffled fur, tachypnea); severe hypothermia as indicated by a temperature of 34°C (∼4.5°C below normal) in the days following bacterial challenge; weight loss equal to 25% of starting weight; and/or severe illness predictive of death or the moribund state. The mice demonstrating extreme signs of illness were immediately humanely euthanized by CO_2_ asphyxiation and cervical dislocation and considered to have succumbed to the infection within 24 hours of achieving aforementioned endpoints. These endpoint criteria have been incorporated to avoid the use of death as endpoint. At the end of experiments, all the remaining mice were also sacrificed. In order to confirm the death of the animals from *S. pyogenes* infections, the spleens of sacrificed mice were isolated and homogenized in PBS. Significant number of *S. pyogenes* CFUs determined by Gram staining, catalase testing, and Lancefield grouping were isolated from these spleens. In contrast, *S. pyogenes* was not isolated from the spleens of survived animals or the animals of control group.

### Statistical analysis

The mice survival data were assessed by the Kaplan-Meier survival curve and the log-rank test. All statistical tests were performed with GraphPad Prism (GraphPad Software Inc.). P values less than 0.05 were considered statistically significant.

## Results

### Inactivation of *rgg2*

The gene encoding Rgg2 (Spy_0496) was identified in all nineteen *S. pyogenes* genomes currently available in public databases. In addition, we amplified a DNA product of the predicted size with PCR using the primers Rgg2-1 and Rgg2+1 in 30 of 30 *S. pyogenes* isolates representing different serotypes (data not shown). The results indicated that *rgg2* is likely to be present in all isolates of *S. pyogenes*. To identify Rgg2-regulated genes and to determine its contribution to virulence, *rgg2* was inactivated in strain SF370 (serotype M1), an isolate from a patient with a wound infection [4]. PCR and nucleotide sequencing confirmed insertional disruption of the *rgg2* locus (data not shown).

### *rgg2* inactivation affects bacterial growth

Inactivation of *rgg2* did not affect cell size, chain length, colony size, the diameter of the zone of hemolysis when cultured with blood agar plates, or the expression of the cysteine proteinase SpeB (data not shown). However, the mutant strain had a shorter lag period and greater growth yield compared to the parental strain when cultured with THY (Fig. 1A). Complementation of the mutant with an intact *rgg2* gene restored the growth yield to that of the wild-type strain SF370 (Fig. 1A). Finally, it was noted that the SF370 strain grew more predominantly at the bottom of the tube compared to the *rgg2* mutant strain (Fig. 1B).

### Rgg2 controls the expression of the Sic protein

**Figure 2:**
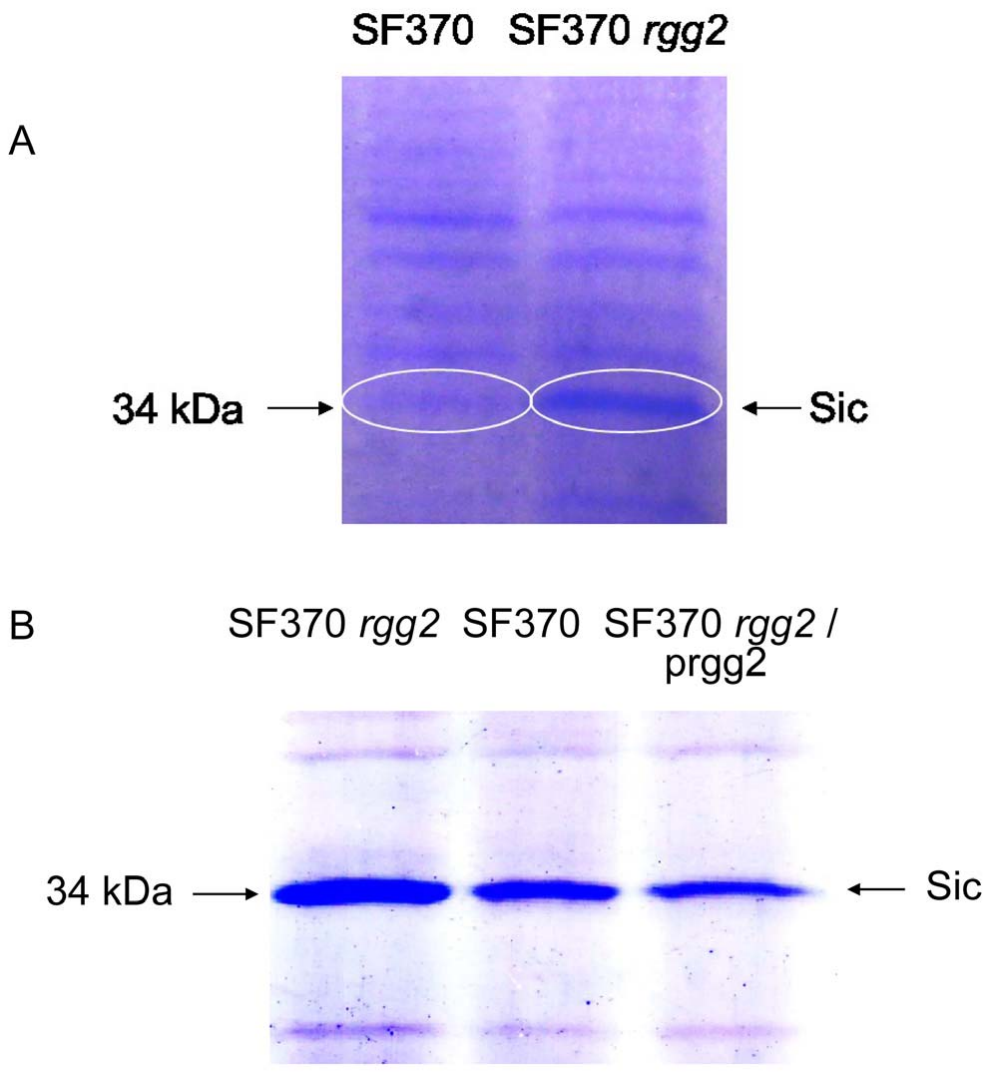
SDS-PAGE analysis of secreted proteins. Panel A. Secreted proteins were collected from middle-exponential phase cultures (SF370 and *rgg2* mutant strains) by acetone precipitation and separated by SDS-PAGE. Both 34 kDa protein bands (indicated with arrow) were excised from the gel and identified as Sic by using mass-spectrometry. Panel B. The production of Sic was restored to the wild-type level by complementation of the *rgg2* mutant with an intact gene expressed by the native promoter.

SDS-PAGE analysis of the proteins secreted by the SF370 and *rgg2* mutant strains revealed a difference in the expression of a 34 kDa protein, which was more abundant in samples from the *rgg2* mutant strain (Fig. 2A). Both bands were analyzed by mass-spectrometry and identified as the streptococcal inhibitor of the complement (Sic). Complementation of the mutant with an intact *rgg2* gene restored SIC production to levels similar to the wild-type strain (Fig. 2B).

### Transcriptome analysis of the wild-type SF370 and SF370 *rgg2* mutant strains

To identify Rgg2-regulated genes, the transcriptomes of SF370 and SF370 *rgg2* were compared in the middle exponential and post-exponential phases of growth with Affymetrix whole-genome chips. In the middle exponential phase of growth, inactivation of *rgg2* altered the expression of 155 genes; transcripts of 86 and 69 genes were more and less abundant, respectively in the mutant stain (Table 2).

**Table 2:** Transcript changes associated with *rgg2* inactivation during the middle-exponential phase of growth*.

Category and SPy no^a^^,^ b	Gene^c^	Description	Fold change(s) (*rgg2*/wt)^d^
**Genes more abundant in the ** ***rgg2*** ** mutant strain**			
**Metabolism**			
1743-1744-1746-1747-1748-1749-1750-1751	*accA/accD/fabZ/accB/fabF/fabG/fabD/fabK*	acetyl-CoA carboxylase subunit alpha, acetyl-CoA carboxylase subunit beta, (3R)-hydroxymyristoyl-ACP dehydratase, acetyl-CoA carboxylase biotin carboxyl carrier protein subunit, 3-oxoacyl-(acyl carrier protein) synthase II, 3-ketoacyl-(acyl-carrier-protein) reductase, putative trans-2-enoyl-ACP reductase II	3.9, 2.4, 2.9, 3.9, 2.6, 4.0, 4.4, 3.3
1753	*acpP*	acyl-carrier-protein S-malonyltransferase	2.7
1754	*fabH*	3-oxoacyl-(acyl carrier protein) synthase III	2.1
2051	-	PTS system, cellobiose-specific IIB component	2.9
2200–2201–2202	*hasA/hasB/hasC*	hyaluronate synthase, UDP-glucose 6-dehydrogenase, UDP-glucose pyrophosphorylase	3.7, 2.6, 2.2
**Stress responsive**			
2077	*csp*	putative cold shock protein	3.7
**Transcription, translation and replication**			
0007–0008	*pth/trcF*	peptidyl-tRNA hydrolase, putative transcription-repair coupling factor	2.3, 2.9
0250	*rpmH*	50S ribosomal protein L34	3.4
0517	*thrS*	threonyl-tRNA synthetase	2.6
0822	*rpmA*	50S ribosomal protein L27	2.2
1234	*rpsT*	30S ribosomal protein S20	2.7
1513	*ileS*	isoleucyl-tRNA synthetase	3.1
1719	*rbfA*	ribosome-binding factor A	2.4
1829	*rpsR*	30S ribosomal protein S18	2.1
1871	*rpsN*	30S ribosomal protein S14	4.0
1955	*rpsO*	30S ribosomal protein S15	2.1
**Transport**			
0137	*atoE*	putative short-chain fatty acids transporter	3.1
0183–0184	*opuAA/opuABC*	putative glycine betaine/proline ABC transporter ATP-binding protein, putative glycine-betaine binding permease protein	2.5, 3.3
0778	-	putative ABC transporter (substrate-binding protein)	2.1
1315–1316	-	hypothetical protein, putative ABC transporter ATP-binding protein	4.1, 4.8
1392	-	putative oxalate formate antiporter	3.0
1728	-	putative ABC transporter (permease)	2.2
2001–2002–2003–2004	*dppB/dppC/dppD/dppE*	transmembrane transport protein, transmembrane transport protein, ATPase protein, ATPase protein	2.1, 2.4, 2.3, 2.3
**Exoproteins/virulence associated**			
0165–0166–0167	*nga/-/slo*	NAD glycohydrolase, hypothetical orf, streptolysin O	8.1, 9.2, 7.0
0861	*mac/ideS*	immunoglobulin G-endopeptidase	2.7
1205	-	putative anti resistance factor	2.5
1436	*mf-3*	putative deoxyribonuclease	2.4
1718	*sse*	secreted esterase	15.7
2009–2010	*-/scpA*	cell surface/fibronectin-binding protein, C5a peptidase precursor	14.6, 11.0
**Hypothetical**			
0105, 0170, 0171, 0210, 0492, 0589, 0793, 0798, 0995, 1036, 1332, 1335, 1437, 1552, 1562, 1610, 1736, 1768, 1832, 1936, 2191	-	hypothetical protein	2.2, 7.4, 4.2, 2.7, 4.1, 2.6, 2.1, 2.6, 5.5, 2.7, 3.1, 2.8, 7.7, 2.0, 2.2, 2.6, 5.6, 2.8, 2.6, 2.2, 6.3
**Miscellaneous**			
0037	*rgg4*	transcriptional regulator, Cro/CI family	8.0
0207	-	putative biotin synthase	2.6
0259	-	putative phosphosugar-binding transcriptional regulator	2.2
0826	*lsp*	lipoprotein signal peptidase	2.3
0841	-	RNA binding protein	2.4
0857	*mur1.2*	putative peptidoglycan hydrolase	2.1
0929	-	putative endonuclease III (DNA repair)	2.2
0971	-	putative terminase, small subunit - phage associated	2.2
0982	-	putative structural protein-phage associated	5.3
1163	*smf*	Smf family DNA processing protein	2.4
1205	*-*	putative anti resistance factor	2.5
1408	*comEC*	putative competence protein	2.6
1615	*comFC*	putative late competence protein	2.4
1698	*glyQ*	glycyl-tRNA synthetase subunit alpha	3.3
1902	*comX1.2*	ComX1-like protein, alternative sigma factor	3.8
2102	-	pseudogene	2.2
2185	*gidA*	tRNA uridine 5-carboxymethylaminomethyl modification enzyme GidA	2.3
2193	-	ABC-type cobalt transport system	2.4
**Genes less abundant in the ** ***rgg2*** ** mutant strain**			
**Metabolism**			
0497–0498	*fpg/coaE*	formamidopyrimidine-DNA glycosylase, dephospho-CoA kinase	−4.4, −4.9
0608	*ppc*	phosphoenolpyruvate carboxylase	−2.2
0629–0630–0631–0632–0634	*agaD/aga/agaV/ugl/agaF*	PTS system, N-acetylgalactosamine-specific IID component, PTS system, N-acetylgalactosamine-specific IIC component, PTS system, N-acetylgalactosamine-specific IIB component, putative unsaturated glucuronyl hydrolase, putative PTS dependent N-acetyl-galactosamine- and galactosamine IIA component	−5.4, −2.7, −3.6, −2.5, −2.9
0830–0831	*pyrR/pyrP*	bifunctional pyrimidine regulatory protein PyrR uracil phosphoribosyltransferase, putative uracil permease	−2.3, −2.0
0900–0901	*pyrF/pyrE*	orotidine 5-phosphate decarboxylase, orotate phosphoribosyltransferase	−5.6, −7.4
1055	*csrA*	methionine sulfoxide reductase B,	−2.4
1057	*-*	PTS system, mannose/fructose family IIA component	−4.1
1058	*-*	PTS system, mannose/fructose family IIB component	−5.5
1059–1060	*−/−*	PTS system, mannose/fructose family IIC component, PTS system, mannose/fructose family IID component	−8.2, −6.5
1111	*-*	putative zinc-containing alcohol dehydrogenase	−2.2
1135	*-*	guanosine 5′-monophosphate oxidoreductase	−4.5
1186–1188–1189–1190–1191	*citD/citE/citF/citX/oadA*	citrate lyase subunit gamma, putative citrate lyase, beta subunit, putative citrate lyase, alpha subunit, 2′-(5″-triphosphoribosyl)-3′-dephospho-CoA: apo-citrate lyase, oxaloacetate decarboxylase	−3.9, −2.9, −2.9,−3.6, −3.3
1204	*guaA*	GMP synthase	−564.1
1541–1542–1543	*arcC/−/−*	carbamate kinase, hypothetical protein, conserved hypothetical integral membrane protein	−9.9,−13.8,−10.1
1544	*arcB*	ornithine carbamoyltransferase	−5.3
1547	*arcA*	arginine deiminase	−2.1
1682–1683–1684	*glpF/glpO/glpK*	putative glycerol uptake facilitator, putative alpha-glycerophosphate oxidase, glycerol kinase	−3.3, −2.9, −2.2
1704–1705–1707–1708	*lacD1/lacC1/lacB.1/lacA.1*	tagatose 1,6-diphosphate aldolase, pseudogene, galactose-6-phosphate isomerase subunit LacB, galactose-6-phosphate isomerase subunit LacA	−7.5, −8.1,−7.4, −8.6
1916–1917–1918–1919–1921–1922	*lacG/lacE/lacF/lacD.2/lacC.2/lacB.2*	6-phospho-beta-galactosidase, PTS system lactose-specific transporter subunit IIBC, PTS system lactose-specific transporter subunit IIA, tagatose 1,6-diphosphate aldolase, tagatose-6-phosphate kinase, galactose-6-phosphate isomerase subunit LacB	−7.7, −5.6, −4.3, −5.0, −3.3, −2.6
2047–2048–2049	*gldA/mipB/pflD*	glycerol dehydrogenase, fructose-6-phosphate aldolase, putative pyruvate formate-lyase 2	−2.7, −3.8, −2.4
**Transport**			
0845	*czcD*	putative cation-efflux system membrane protein	−4.0
0904	-	putative ABC transporter (permease)	−3.0
1018–1019	-	putative ABC transport protein (permease), ABC transporter ATP-binding protein	−2.4, −2.2
1131	-	Na^+^ driven multidrug efflux pump	−2.6
1714–1715–1717	*copZ/copA/copY*	putative copper chaperone-copper transport operon, putative cation-transporting ATP-ase - copper transport operon, putative negative transcriptional regulator	−10.9, −8.2, −2.0
**Exoproteins/virulence associated**			
1008	*speH*	streptococcal exotoxin H precursor	−2.0
**Regulatory**			
0496	*rgg2*	putative positive transcriptional regulator	−2.3
0533	-	putative positive regulator	−2.6
**Hypothetical**			
0238, 0550–0552, 1017, 1203, 1340, 1603–1604, 2172–2173	-	hypothetical protein	−2.3, −2.8, −2.5, −2.5, −5.7, −10.9, −2.7, −4.1,−2.5, −2.3
**Miscellaneous**			
0470	-	myosin-cross-reactive antigen	−2.0
0902	*amiC*	amidase	−3.2

*Based on 2-fold difference or P<0.05.

a SPy numbers designate open reading frames based on the SF370 *S. pyogenes* annotation [4].

b Contiguous genes likely to be cotranscribed are separated with a dash.

c Hyphen indicates an unnamed gene.

d Change in transcript level for *rgg2* mutant compared to that for the wild type.

Gene transcripts associated with purine, pyrimidine, and lactose metabolism were less abundant in the mutant strain (Table 2). Specifically, there were decreases in expression of four genes involved in pyrimidine metabolism (SPy_830, 831, 0900, 0901) with an average decrease of 4.2-fold compared to the wild-type strain. There were two differences in expression of genes associated with purine metabolism (Spy_1135, 1204) with a 4.5 and 564-fold decrease in expression in the mutant, respectively. The difference in *guaA* (Spy_1204) expression was the largest difference detected. There were ten differences in the expression of genes associated with lactose metabolism (SPy_1704, 1705, 1707, 1708, 1916, 1917, 1918, 1919, 1921, 1922) with an average decrease in the mutant strain of 6-fold. Finally, genes of the arginine deaminase pathway (SPy_1541, 1542, 1543, 1544, 1547), which convert arginine to ATP and ornithine, were expressed an average of 8.2-fold less in the mutant (Table 2).

Among genes that were expressed at a higher level in the mutant were several involved in replication, transcription, and translation processes, although these were expressed an average of only 2.7-fold higher in the mutant (Table 2).

Also noteworthy was the elevated expression in the mutant strain of three genes associated with competence for DNA transformation including the alternative sigma factor ComX1.2 and two late competence genes (ComEC and ComFC; Table 2).

Finally, inactivation of *rgg2* increased by an average of 8-fold the expression of known virulence associated genes including *sse* (secreted streptococcal esterase), *scpA* (C5a peptidase), *slo* (streptolysin O), *nga* (NAD-glycohydrolase), and *mf*-3 (secreted DNAse) (Table 2). In addition, transcripts encoding the enzymes necessary for the synthesis of the hyaluronic acid capsule, HasABC, were 2.8-fold higher in the mutant.

**Figure 3:**
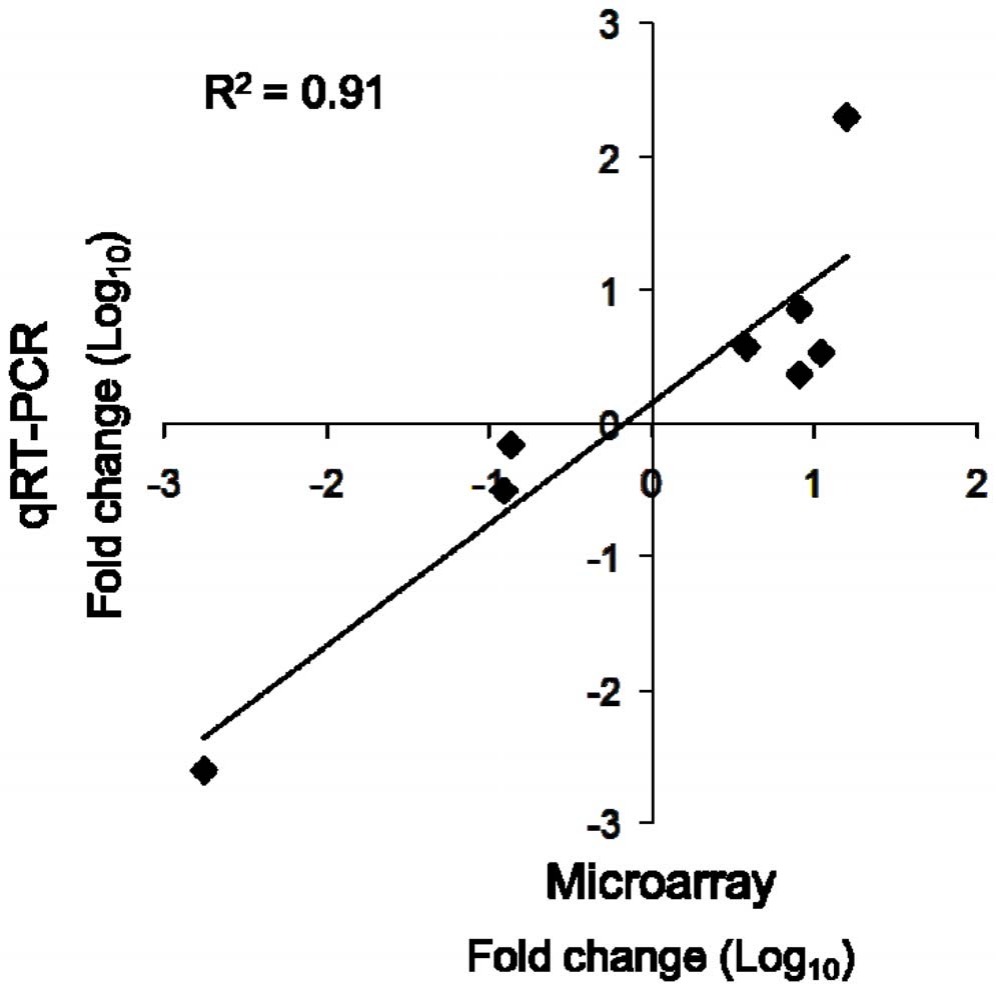
Correlation between results obtained with DNA microarrays and qRT-PCR. Each point represents the difference in transcript levels of selected genes in *rgg2* mutant strains in comparison with wild-type strains as determined by DNA microarrays (Y-axis) and qRT-PCR (X-axis).

We also measured the expression of selected genes using quantitative RT-PCR (qRT-PCR). Transcripts of Spy_0037, Spy_1902, Spy_0165, Spy_1718, and Spy_2010 were increased in the mutant while those of Spy_0901, Spy_1059, and Spy_1204 were decreased (Table 1), which was consistent with the results obtained with DNA microarrays, R^2^ = 0.91 (Fig. 3). The magnitude of the changes varied between results obtained with qRT-PCR compared to arrays, probably because RNA samples used for qRT-PCR were isolated independently from those used for the array analysis. Also of note, qRT-PCR analysis indicated that *guaA* transcripts were not present at detectable levels in the *rgg2* mutant strain (the Ct values were similar in reactions containing reverse transcriptase or not); in contrast, expression levels were relatively abundant in the wild-type strain.

In contrast to the exponential phase of growth, differences in the expression of only 14 genes were identified in the post-exponential phase of growth and all were expressed less in the mutant strain (Table 3). Based on genome annotation, half of these are involved with pyrimidine metabolism and were expressed 6 to 10-fold lower in the mutant strain. Similar to results obtained in the exponential phase, the largest difference in the post-exponential phase was a decrease in *gua* expression in the mutant strain.

**Table 3:** Transcript changes associated with *rgg2* inactivation during the post-exponential phase of growth*.

Category and SPy no^a^^,^ b	Gene^c^	Description	Fold change(s) (*rgg2*/wt)^d^
**Metabolism**			
0497–0498	*fpg/coaE*	formamidopyrimidine-DNA glycosylase, dephospho-CoA kinase	−6.4, −9.1
0830–0831–0832–0833–0835	*pyrR/pyrP/pyrB/carA/carB*	bifunctional pyrimidine regulatory protein PyrR uracil phosphoribosyltransferase, putative uracil permease, aspartate carbamoyltransferase catalytic subunit, carbamoyl phosphate synthase small subunit, carbamoyl phosphate synthase large subunit	−17.8, −13.5, −16.4,−19.3, −18.5
0900–0901	*pyrF/pyrE*	orotidine 5-phosphate decarboxylase, orotate phosphoribosyltransferase	−6.0, −9.9
1204	*guaA*	GMP synthase	−1025.0
1991	*trpG*	anthranilate synthase component II	−2.5
**Transport**			
0903–0904	-	putative ABC transporter (binding protein), putative ABC transporter (permease)	−6.1, −4.9
**Regulatory**			
1870	-	GntR family transcriptional regulator	−2.4

*Based on 2-fold difference or P<0.05.

a SPy numbers designate open reading frames based on the SF370 *S. pyogenes* annotation [4].

b Contiguous genes likely to be cotranscribed are separated with a dash.

c Hyphen indicates an unnamed gene.

d Change in transcript level for *rgg2* mutant compared to that for the wild type.

### Rgg2 regulation influences biofilm formation

**Figure 4:**
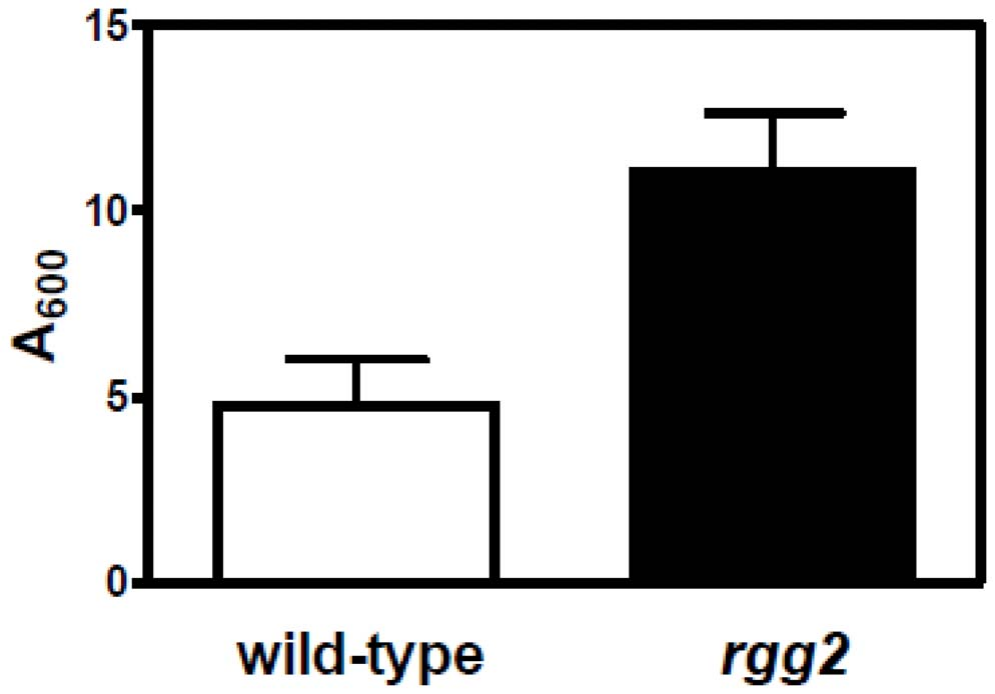
Enhanced static biofilm formation in the *rgg2* mutant strain. Biofilm formation was compared between the wild-type and *rgg2* mutant strains. The strains were cultured with CDM and static biofilm formation determined after 24 hrs incubation. The results are shown as the means and SEMs from three independent experiments. The difference between the strains was statistically significant (P<0.05).

Members of the Rgg family of regulators are often important in biofilm formation in *S. pyogenes* [3] and in related bacteria such as *S. gordonii* [21] and *S. pneumoniae* [7], among others. Therefore, we compared biofilm formation between the wild-type SF370 strain and the *rgg2* mutant strain in CDM with, and without, the additional of exogenous peptides (neopeptone). Inactivation of *rgg2* in strain SF370 increased biofilm formation compared to the parental strain (Fig. 4). We also found that the addition of peptides to CDM abrogated biofilm formation in both strains (data not shown).

### Rgg2 contributes to the virulence of *S. pyogenes*

To determine if Rgg2 influences virulence, we used three approaches. First, we assessed adherence to epithelial cells as described in Materials and Methods. As result, the adhesion indexes of SF370 and *rgg2* mutant strains to single epithelial cell respectively were as follows: *i*) donor #1; 103±23 and 39±12; *ii*) donor #2; 26±3 and 8±2; *iii*) donor #3; 32±8 and 10±4. Together, the adherence capacity of the *rgg2* mutant was about 3-fold less compared to the SF370 wild-type strain.

**Figure 5:**
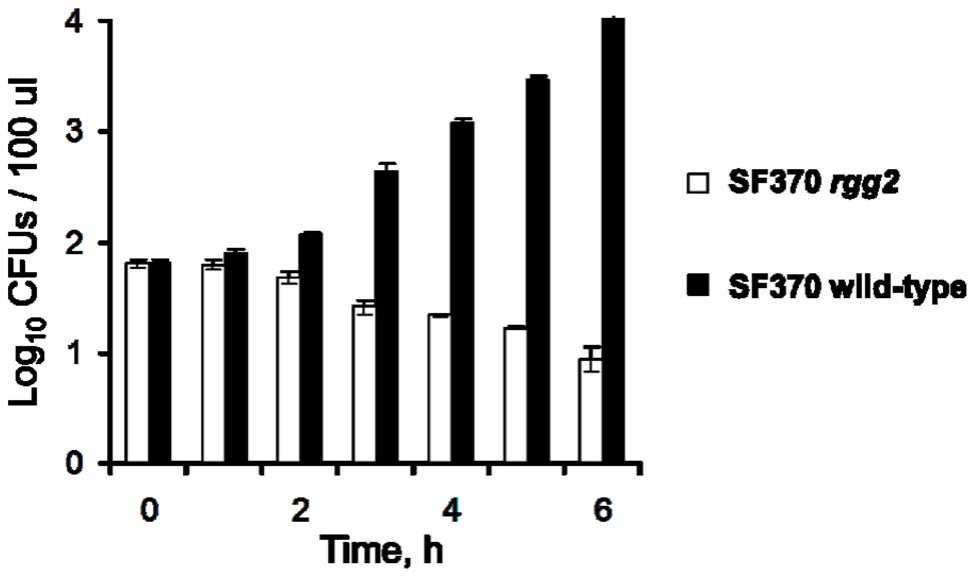
Survival assay of *S. pyogenes* strains in the whole human blood. 450 CFUs of wild-type strain SF370 and the *rgg2* mutant were independently mixed with heparinized human blood and incubated at 37°C. The number of viable CFUs were determined every hour by the plating aliquots onto solid agar media. The results are shown as the means and SEM from three independent experiments.

**Figure 6:**
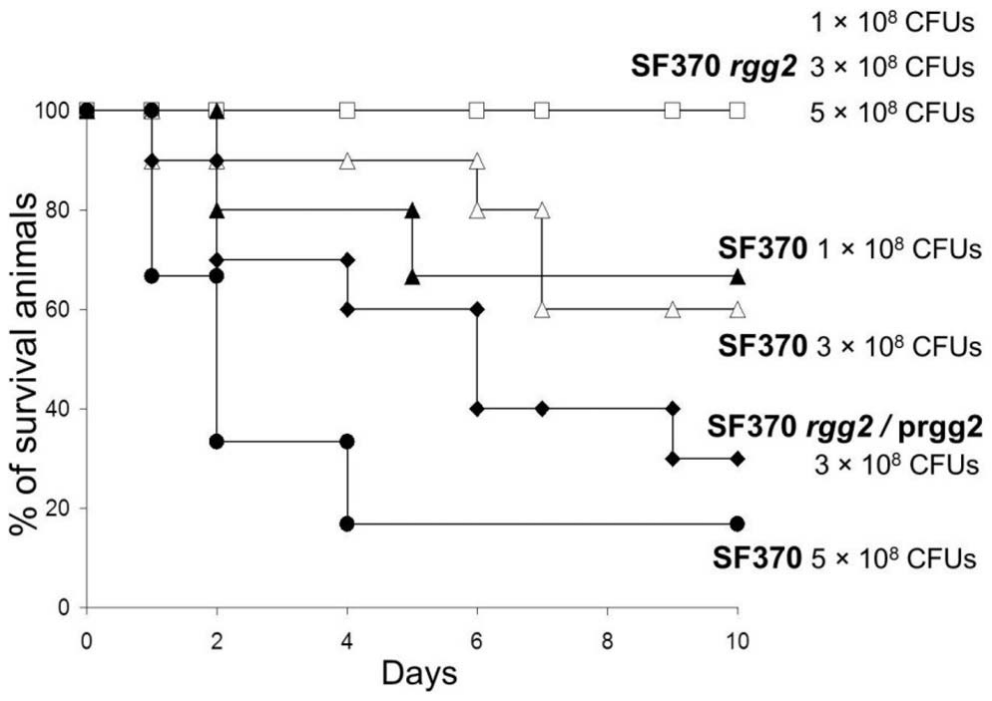
Murine intraperitoneal *S. pyogenes* infection. Experimental groups of animals were infected with different doses of SF370, *rgg2* mutant and SF370 *rgg2*/prgg2. Mice were observed for 10 days. X-axis indicates number of the days after infection. Y-axis indicates percentage of surviving mice.

In addition, the mutant strain was more sensitive to killing when incubated with whole human blood compared to the wild-type strain (Fig. 5). Finally, all mice infected with the *rgg2* mutant (1–5×10^8^ CFUs per animal) survived whereas only 17% of mice inoculated with the SF370 parental strain (5×10^8^ CFUs per animal) survived (Fig. 6). Importantly, complementation of the mutant restored the virulence associated with SF370 (Fig. 6). Together, the results suggest that Rgg2 is essential for the virulence of SF370.

### The attenuated *rgg2* mutant strain protects mice against challenge with wild-type *S. pyogenes*

**Figure 7:**
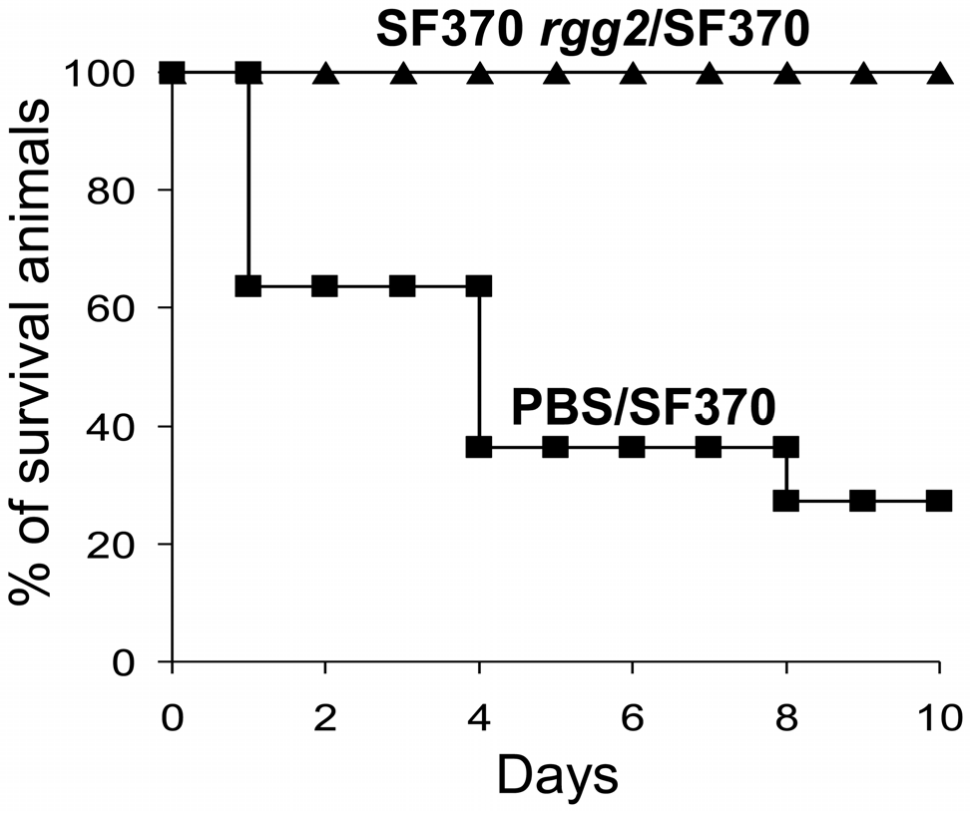
Protection of mice against lethal *S. pyogenes* infection. Two experimental groups of animals were infected with the avirulent SF370 *rgg2* mutant strain (4×10^8^ CFUs per animal) or PBS as a control. After 19 days, the mice in both groups were challenged with the wild-type SF370 strain (3×10^8^ CFUs per mouse) and monitored 10 days. The X-axis indicates number of the days after infection with wild-type SF370 and the Y-axis indicates percentage of surviving mice.

To determine if the avirulent SF370 *rgg2* mutant strain could protect mice against subsequent challenge with the parental strain, mice were inoculated intraperitoneally with the mutant strain (4×10^8^ CFUs per animal), or PBS as a control. As expected, all the animals survived. After 19 days, mice in both groups (inoculated with either the SF370 *rgg2* mutant strain or PBS) were challenged with the wild-type SF370 strain (3×10^8^ CFUs per animal). 73% of mice that received prior inoculation with PBS died, while none of the mice previously inoculated with the avirulent mutant strain died (Fig. 7). The results show that prior exposure to the attenuated *rgg2* mutant strain can protect mice against an otherwise lethal *S. pyogenes* infection.

## Discussion

Among various species of the Firmicutes family possessing *rgg* genes, the regulators play crucial roles in controlling a variety of important processes including metabolism, biofilm formation, and natural competence [3], [7]–[10], [13], [18], [21], [22]. Here, we set out to identify the regulon of Rgg2 in *S. pyogenes* and to determine its contribution to virulence. To do so, we used directed mutagenesis, transcriptome profiling, *ex vivo* measures of virulence, and a murine model of bacteremia. We discovered that Rgg2 controls the expression of dozens of genes in rich media primarily in the exponential phase of growth, including genes associated with nucleotide metabolism and virulence. We also found that Rgg2 contributes to virulence based on the finding that the mutant was unable to grow in human blood or cause mortality in mice. Finally, we showed that mice inoculated with the avirulent *rgg2* mutant were protected from disease following challenge with parental isolate. Overall, the results contribute to a growing body of evidence that the members of the Rgg family of transcriptional regulators are critically important in the control of gene expression, fitness, and virulence in *S. pyogenes* and related species.

The *rgg2* mutant was associated with an increase in the expression of virulence-associated genes (*nga*, *slo*, *mf-3*, *scpA*, and *sse*), which suggested that the mutant might be more virulent; however, in contrast to the parental isolate, the mutant was unable to grow in human blood *ex vivo* or cause mortality in mice (Figs. 5, 6A, 6B). There are a several possible explanations for the ostensibly contradictory findings. First, in addition to regulating virulence gene expression, directly or indirectly, Rgg2 also controls the expression of several metabolic genes, which are likely to be important in adapting to, and surviving in, different environmental conditions such as rich media, human and murine blood, and various murine tissues. Indeed, as we previously reported that inactivation of *rgg1* resulted in an inability of *S. pyogenes* to utilize sucrose as the sole carbon and energy source [13]. Second, it remains unknown if elevated expression of virulence genes in rich media also occurs during infection of mice. Third, the outcome of infection is a complex process involving dynamic interactions among multiple host and bacterial factors [1]. Thus, overall it seems likely that inactivation of *rgg2* hinders the ability of the bacteria to coordinate gene expression in the host and this dysregulation decreases the fitness of the microbe and its ability to survive in human blood or persist in mice and cause disease.

The chromosome of *S. pyogenes* encodes the proteins required for natural DNA transformation, including an alternate sigma factor (ComX1.2) and late competence genes [4]. In addition, the expression of competence genes is controlled by hydrophobic peptides (quorum sensing) and members of the Rgg family [5], [9]. For example, Rgg4 (ComR) is an Rgg regulator that is required for expression of the late competence genes in *S. pyogenes* [9]. Here, we found that Rgg2 represses Rgg4 by 8-fold. Thus, it seems likely that increased Rgg4 in the *rgg2* mutant is responsible for the increased expression of the alternative competence associated sigma factor gene *comX1.2* (Spy_1902), competence genes *comEC* (Spy_1408) and *comFC* (Spy_1615); although we can not rule out the possibility that Rgg2 also directly binds to competence genes to influence expression levels.

A comparison of the Rgg1 [23] and Rgg2 regulons (Tables 2 and 3) during the post-exponential phase of growth of strain SF370 did not reveal any similarities. In contrast, several genes were similarly regulated by Rgg1 during post-exponential growth and by Rgg2 during middle-exponential growth. They included increases in *rgg3* (SPy_0037) expression by 3-fold and 8-fold in the post-exponential cultures of *rgg1* and exponential cultures of *rgg2* mutants, respectively; increases in the secreted esterase Sse (SPy_1718) by 2-fold and 15.7-fold; and decreases in a hypothetical protein (SPy_1017) by -3-fold and -2.5-fold, respectively. Together, these data support the idea of cross-talk among different Rgg paralogues [3] which may work to fine-tune expression levels in response to various signals.

In several pathogens, quorum sensing plays an important role in controlling the formation of biofilms and their dispersal [24]. Rgg1, Rgg2, and Rgg3 can all affect biofilm formation in *S. pyogenes*. Single gene deletion of the regulatory genes in a serotype M49 can promote (Rgg1), diminish (Rgg2) or have relatively little affect (Rgg2) on abiotic biofilm formation [3]. Moreover, *rgg2* deletion in an *rgg1* mutant ablates the increase in biofilm observed when only *rgg1* is inactivated, perhaps indicating a hierarchy of regulatory control [3]. Using the serotype M1 strain SF370, we observed an increase in abiotic biofilm formation in the *rgg2* mutant when cultured with peptide free CDM; however, when peptides were present there was no difference in biofilm formation and neither strain produced a substantial biofilm although there was a marked increase in planktonic growth. The secreted peptides involved in Gram-positive quorum sensing, including Rgg transcriptional regulators, are imported to the cytoplasm by the oligopeptide permease system (Opp) [25]. In some signaling systems, peptides present in rich media are thought to compete for Opp-mediated transport, which can dampen the quorum-sensing specific responses. While disruption of peptide-mediated signaling via Rgg2 by the addition of neopeptone may explain the abrogation of biofilm formation in peptide-supplemented media, additional study is needed to determine the molecular basis for the observation.

GMP synthase (GuaA, SPy_1204) is the final enzyme in both salvage and *de novo* pathways of guanine synthesis. It is essential for virulence in a number of different bacterial pathogens including *Borrelia burgdorferi* [26], *Salmonella typhi* [27], and *Francisella tularensis* [28]. Additional studies are needed to determine the extent to which Rgg2 regulation of *guaA* expression contributed to its inability to grow in human blood or cause mortality in mice.

In conclusion, we found that Rgg2 controls gene expression in both the exponential and post-exponential phases of growth in rich media, although more genes are controlled in the exponential phase. The targets of regulation included genes involved in nucleotide metabolism, DNA transformation, and virulence. The *rgg2* mutant was avirulent in mice and could be used to protect mice challenged with the virulent wild-type strain. The results contribute the growing body of evidence illustrating the importance of the family of Rgg regulatory proteins.
